# Effects of IncobotulinumtoxinA in the Infraorbital Nerve Chronic Constriction Injury Model of Trigeminal Pain in Rats

**DOI:** 10.3390/biomedicines14051175

**Published:** 2026-05-21

**Authors:** Wojciech Danysz, Paulina Nunez-Badinez, Andreas Gravius, Klaus Fink, Jens Nagel

**Affiliations:** Merz Therapeutics GmbH., Eckenheimer Landstraße 100, 60318 Frankfurt am Main, Germany; wojciech.danysz@merz.de (W.D.); andreas.gravius@merz.de (A.G.); klaus.fink@merz.de (K.F.); jens.nagel@merz.de (J.N.)

**Keywords:** botulinum toxin, botulinum toxin A, duration of action, incobotulinumtoxinA, infraorbital nerve chronic constriction injury, NT 201, preventive, therapeutic

## Abstract

**Background/Objectives**: Trigeminal neuralgia (TN) is a debilitating neurological condition characterized by recurrent, severe pain linked to peripheral and central sensitization within trigeminal pathways. Current pharmacologic treatments are limited by inadequate efficacy or dose-limiting side effects, and botulinum neurotoxin type A (BoNT/A) has emerged as a viable option. However, its potential use in the management of TN is hampered by methodological limitations in existing studies and a lack of pivotal clinical trials. This study investigated the efficacy, optimal treatment site, preventive utility, and duration of effect of incobotulinumtoxinA (Inco/A), a BoNT/A, in a model of TN. **Methods**: An infraorbital nerve chronic constriction injury model was used to induce mechanical allodynia in male Sprague–Dawley rats, reproducing the trigeminal sensitization seen in TN. The effects of subcutaneous Inco/A (1, 2, and 4 U) were measured using the mechanical sensitivity (von Frey) test to evaluate the dose response, effect of injection location, potential preventive nature of treatment, and duration of benefit. **Results**: Inco/A produced a robust, dose-dependent reduction in mechanical allodynia, predominantly via a local mechanism of action. Both preventive and therapeutic administration of Inco/A was efficacious, with significant reduction in allodynia even when administered up to 28 days before nerve injury. The anti-allodynic effect persisted up to 56 days post-injection. **Conclusions**: Inco/A is highly effective in alleviating mechanical allodynia in a validated rat model of TN. The findings highlight Inco/A as a promising candidate for clinical translation in TN and related neuropathic pain syndromes and support systematic investigation in well-controlled human trials.

## 1. Introduction

Trigeminal neuralgia (TN) is a neuropathic facial pain syndrome characterized by sudden, severe, electric shock-like or stabbing unilateral pain that occurs in the distribution of one or more branches of the trigeminal nerve [1]. Individual attacks usually last from a fraction of a second to 2 min but often recur in rapid succession and can occur up to hundreds of times per day [2]. There are three main types of TN: classical TN is the most common and results from neurovascular compression of the trigeminal nerve root; secondary TN is associated with underlying pathology, such as multiple sclerosis (MS) or intracranial tumors; and idiopathic TN has no identifiable cause [3]. The lifetime prevalence of TN in the general population is relatively low (0.11% globally), but it is markedly more prevalent in patients with MS (1–2%), and TN presentation precedes a diagnosis of MS in 15% of cases [4,5,6].

TN is a highly debilitating disorder, with a multifaceted impact on basic daily functions such as speaking, eating, drinking, facial hygiene, and social interaction, and it frequently triggers comorbid psychiatric disorders, particularly depression and anxiety [7]. TN can therefore result in high disability rates and a substantially diminished quality of life (QoL) [2,8]. First-line pharmacological treatment consists of carbamazepine or oxcarbazepine, with second-line options including gabapentin, lamotrigine, and baclofen [9]. Intravenous fosphenytoin or lidocaine provides acute rescue therapy for severe exacerbations. However, despite these treatment options, many patients with TN develop refractory pain. In addition, intolerable medication-related side effects that include dizziness, cognitive impairment, nausea, electrolyte abnormalities, and severe dermatological reactions may necessitate drug dose reductions or discontinuations, potentially limiting long-term therapeutic efficacy [2,10,11]. When combined with the disabling nature of TN, this therapeutic gap represents a significant unmet medical need.

TN shares many clinical and pathophysiological features with migraines, with both conditions involving activation and sensitization within the trigeminal nociceptors (peripheral sensitization) as well as dysregulation of central pain processing pathways (central sensitization) [12,13]. Moreover, the risk of developing TN is higher in those affected by migraines [14]. Therefore, therapies targeting migraines may offer therapeutic benefit for both conditions. Botulinum neurotoxin type A (BoNT/A) is particularly promising in this context because of its established efficacy in chronic migraines (PREEMPT 1 [NCT00156910] and 2 [NCT00168428]) and its direct actions on trigeminal nociceptive pathways [15,16]. Further clinical trials are currently under way to evaluate the safety and efficacy of BoNT/A in both chronic and episodic migraines (MINT-C [NCT07018713] and MINT-E [NCT07018700]).

Systematic reviews of clinical trials and case reports indicate that off-label use of BoNT/A for the treatment of medically refractory TN is both safe and efficacious [17,18]. However, most existing studies have methodological limitations and therefore do not meet the requirements for regulatory approval. Additionally, important gaps remain regarding optimal dosing regimens, long-term safety and efficacy profiles, mechanism of action, and performance compared with standard TN therapies [19].

The infraorbital nerve chronic constriction injury (IoN-CCI) is a well-established rat model of trigeminal pain that produces mechanical hyperalgesia and tactile allodynia in the vibrissal pad territory innervated by the injured nerve [20,21] as well as peripheral trigeminal sensitization [22]. BoNT/A has previously been shown to partially reverse facial mechanical allodynia and hyperalgesia in the IoN-CCI rat model, without affecting motor coordination [23,24,25]. BoNT/A also normalized the expression of ion channels related to neuronal excitability and decreased neuronal responses in the trigeminal nociceptive pathway [23,24,25].

IncobotulinumtoxinA (Inco/A; Xeomin^®^ [Merz Therapeutics, Frankfurt am Main, Germany, research code NT 201]) is a highly purified BoNT/A formulation. Unlike conventional BoNT/A products, it has a minimized protein load and the highest specificity among existing BoNT/A products, a feature that may decrease immunogenicity and the risk of neutralizing antibody formation [26]. Inco/A is approved for clinical use as Xeomin^®^ across multiple indications [27].

The objectives of the current series of studies were to investigate aspects of the anti-allodynic effect of BoNT/A treatment in TN that are currently weak or entirely absent, including several aspects of Inco/A’s anti-allodynic mode of action, using an IoN-CCI rat model of TN. These included the site of action, the potential preventive nature of treatment and the duration of action of Inco/A. The optimal therapeutic dose of Inco/A in this rat model was also investigated.

## 2. Materials and Methods

All studies were performed at HD Bioscience (HDB), Shanghai, China (now WuXi Biology, WuXi AppTec, Shanghai, China). This facility has full Association for Assessment and Accreditation of Laboratory Animal Care international accreditation. Study protocols were reviewed and approved by the HDB Institutional Animal Care and Use Committee before study initiation. This study adhered to the ARRIVE 2.0 guidelines (Essential 10 and Recommended Set) for reporting animal research [28]. The animal study protocol was approved by the Institutional Review Board of HD Bioscience (HDB), now WuXi Biology, WuXi AppTec, Shanghai, China (protocol code AUF#108, approved on 10 August 2020).

### 2.1. Animals

Male Sprague–Dawley rats (weighing 200–230 g) were obtained from Beijing Vital River Laboratory Animal Technology Co. Ltd. (Charles River Laboratoires, Beijing, China) and acclimatized to the study facility for 1 week before study initiation. Rats were housed in groups of four to five per cage (42 × 31 × 18 cm) with sterile bedding, ad libitum access to purified water, and standard certified laboratory rodent chow (Beijing Keao Xieli Feed Co., Ltd., Beijing, China) throughout the study.

All experimental groups were handled following the same standardized procedures and timelines. Group handling order was consistent across experimental conditions to minimize variability, with each animal participating in only one study. Animals and cages were kept in the same locations throughout the study. Animals were acclimatized to the testing room for at least 10 min before each session. Animal health and welfare were monitored daily by a qualified veterinarian who looked for signs of distress, including altered grooming, excessive licking or scratching, impaired locomotion, feeding difficulties, or other abnormal behaviors indicative of reduced well-being. Body weight and health status were monitored biweekly throughout the study as a humane endpoint. All studies were designed and conducted in accordance with the 3R principles, aiming to replace the use of animals where possible, reduce the number used, and refine procedures to minimize animal suffering, in line with ethical standards. All procedures were conducted with systematic efforts to minimize suffering and distress. At the end of the study, animals were euthanized by carbon dioxide inhalation in accordance with the American Veterinary Medical Association guidelines for the euthanasia of animals (2020 edition).

### 2.2. Induction of Neuropathic Trigeminal Pain

All studies involved generation of neuropathic trigeminal pain via surgically induced IoN-CCI. Using a sterile technique and under general anesthesia with intramuscular xylazine, tiletamine, and zolazepam, a 1 cm intraoral incision was made along the gingivobuccal margin proximal to the first molar, allowing the facial hair and vibrissae to remain anatomically intact. Approximately 0.5 cm of the infraorbital nerve was isolated from surrounding tissue, and two loose ligatures of 4-0 chromic gut were placed circumferentially around the nerve without completely occluding the lumen. The incision was closed using three interrupted 4-0 silk sutures. INOHV IODPHOR disinfecting solution and ANNJET 75% ethanol disinfectant were used to ensure that the wound remained aseptic. For the sham-operated group, the surgical procedure was identical, except the nerve was not ligated. In all studies, the day of surgery was designated as day 0.

### 2.3. Study Treatments

Rats that had undergone IoN-CCI received no further treatment or were subcutaneously injected once with 5 µL Inco/A or vehicle (normal saline [0.9% sodium chloride]); Inco/A was reconstituted and made up to this volume using vehicle. The amount of Inco/A injected, the timing of injections relative to surgery, and the injection site varied by study (see Section 2.5). The majority of the injections were administered into one vibrissal pad. For these injection sites, the terms ‘ipsilateral’ and ‘contralateral’ refer to the injection site’s relationship to the site of the IoN-CCI procedure.

### 2.4. Assessment of Facial Mechanical Allodynia

Facial vibrissal pads were gently stimulated with a plastic stick at 30 s intervals for 10 min (uncalibrated force within the range of the monofilaments) to habituate animals to the procedure and proximity of the apparatus. Mechanical sensory testing was performed using von Frey monofilaments (North Coast Medical Inc., Morgan Hill, CA, USA) when rats were in a quiescent state (limbs in contact with the ground and no spontaneous locomotion, tremor, grooming, or active sniffing behavior). Monofilaments with a range of standardized bending forces were available for use (Table 1). Monofilaments ranging from 0.16 g to 15.0 g were used (see below).

Von Frey monofilaments were applied to the whisker pad region ipsilateral to the induced IoN-CCI. Responses were considered positive if any of the following behaviors occurred: rapid head withdrawal accompanied by face washing of the stimulated facial area; escape, postural curling, head hiding, or biting/grasping of the stimulus; or asymmetric face grooming, comprising an uninterrupted series of three or more face-wash strokes directed exclusively to the stimulated facial area. Testing commenced with the 2.0 g filament. If a positive response was observed, the next weakest filament was applied, and if a negative response was observed, the next strongest filament was applied. Testing continued until five filaments had been applied. The final mechanical withdrawal threshold (MWT, measured in grams-force) was determined by adding 0.5 to the final filament number if there had been no response to this final filament or subtracting 0.5 from the final filament number if there had been a positive response. The MWT was calculated using the following formula:

MWT (grams-force) = $10^{\left(x\times F+B\right)}$, where *x* is 0.182, *F* is the filament number, and *B* is −1.47 [29].

The individuals performing the behavioral assessments were blinded to group allocation (IoN-CCI or sham) and treatment (Inco/A, vehicle or no treatment).

### 2.5. Study Designs

**Figure 1:**
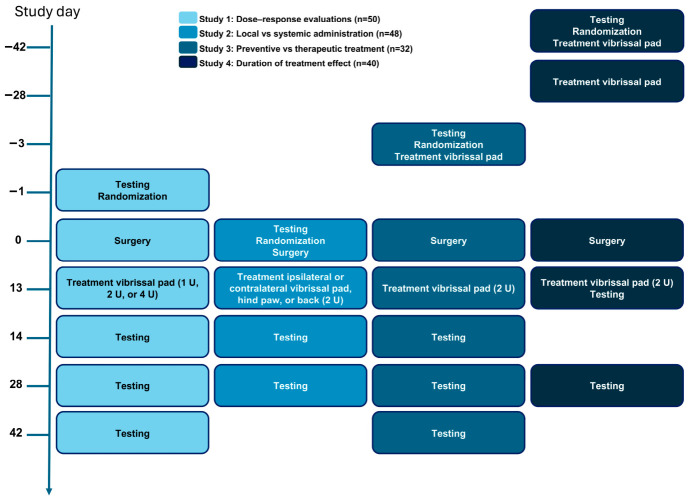
Study design. Study 1: IncobotulinumtoxinA (Inco/A) was injected into the ipsilateral vibrissal pad* on day 13, and mechanical withdrawal threshold (MWT) was assessed on days 14, 28, and 42. Study 2: Inco/A was injected into the ipsilateral vibrissal pad*, contralateral vibrissal pad*, hind paw, or back on day 13, and MWT was assessed on days 14 and 28; Study 3: Inco/A was injected into the ipsilateral vibrissal pad on day −3 or day 13, and MWT was assessed on days 14, 28, and 42; Study 4: Inco/A was injected into the ipsilateral vibrissal pad on days −42, −28, or 13, and MWT was assessed on days 13 and 28. The studies included the following control groups: sham-operated group (all studies) and infraorbital nerve chronic constriction injury (IoN-CCI) vehicle-treated animals (study 1) or IoN-CCI animals without treatment (studies 2–4). * Contralateral vibrissal pad: vibrissal pad contralateral to the injured nerve/site of sham surgery; ipsilateral vibrissal pad: vibrissal pad ipsilateral to the injured nerve/site of sham surgery.

The four sub-studies reported herein (Figure 1) were designed to determine the optimal therapeutic dose (study 1), effect of local versus systemic treatment (study 2), preventive versus therapeutic treatment (study 3), and duration of anti-allodynic effect (study 4). In each study, rats were individually randomized to experimental groups at baseline and stratified by body weight to ensure balanced distribution across treatment conditions. All sub-studies included an additional two groups to control for the effect of surgery (sham-operated group) and treatment (vehicle-treated or no-treatment group, depending on the study). In addition to the post-surgical MWT assessment sessions specified below, sensory responses were tested at baseline (before model induction) in study 1.

#### 2.5.1. Study 1: Inco/A Administration and Evaluation of Dose–Response

Inco/A (1, 2, or 4 U) was injected into the ipsilateral vibrissal pad of rats on day 13. The study also included sham-operated and IoN-CCI vehicle-treated animals. Facial mechanical allodynia was assessed in all five groups (*n* = 10 per group) on days 14, 28, and 42 (1, 15, and 29 days post-treatment).

#### 2.5.2. Study 2: Effects of Local Versus Systemic Inco/A Administration

Inco/A (2 U) was injected into the ipsilateral vibrissal pad, the contralateral vibrissal pad, the hind paw, or the back of rats on day 13. The study also included sham-operated and IoN-CCI animals without treatment. Facial mechanical allodynia was assessed in all six groups (*n* = 8 per group) on days 14 and 28 (1 and 15 days post-treatment).

#### 2.5.3. Study 3: Effects of Preventive Versus Therapeutic Inco/A Administration

Inco/A (2 U) was injected into the ipsilateral vibrissal pad of rats on day −3 or day 13. The study also included sham-operated and IoN-CCI animals without treatment. Facial mechanical allodynia was assessed in all four groups (*n* = 8 per group) on days 14, 28, and 42 (1, 15, and 29 days post-treatment).

#### 2.5.4. Study 4: Duration of Inco/A Treatment Effect

Inco/A (2 U) was injected into the ipsilateral vibrissal pad of rats on days −42, −28, or 13. The study also included sham-operated and IoN-CCI animals without treatment. Facial mechanical allodynia was assessed in all five groups (*n* = 8 per group) on days 13 or 28 (i.e., up to 70 days post-treatment).

### 2.6. Statistical Methods and Figure Generation

An a priori power analysis performed by HDB based on their historical data and published data from the established IoN-CCI model [19,20,23,24] indicated that a minimum sample size of 10 rats per group would be required to demonstrate a statistically significant between-group difference in MWT with a statistical power of 0.8 at an alpha level of 0.05.

Tactile stimuli follow Weber’s law and are sensed on a log scale [30]. MWT values were therefore log_10_-transformed before the statistical analysis using GraphPad Prism (version 10, GraphPad Software LLC, Boston, MA, USA). Treatment groups were compared using one-way analysis of variance (ANOVA) followed by Tukey’s multiple comparisons test or repeated measures two-way ANOVA with Geisser–Greenhouse correction (if applicable), followed by Tukey’s multiple comparisons test. Results are expressed as mean ± standard error of the mean (SEM). Each animal was considered an experimental unit. No animals or data points were excluded from the analysis.

In study 4, an additional analysis of the relationship between the timing of Inco/A administration and sensory testing was evaluated by analyzing normalized MWTs, with sham-operated values designated as 100% (indicative of no pain; lower percentages reflect greater allodynia).

Figure 1 was created using Microsoft^®^ PowerPoint^®^ for Microsoft 365 MSO (Version 2603, Microsoft Corporation, Redmond, WA, USA). All remaining figures were created using GraphPad Prism (version 10, GraphPad Software LLC, Boston, MA, USA).

## 3. Results

Across all studies, no post-surgical complications were observed and no adverse events were observed; no animals or data points were excluded during data analysis. Changes in body weight for each of the study groups are provided in Supplementary Tables S1–S4.

### 3.1. Study 1: Dose–Response Relationship and Anti-Allodynic Effects of Inco/A

**Figure 2:**
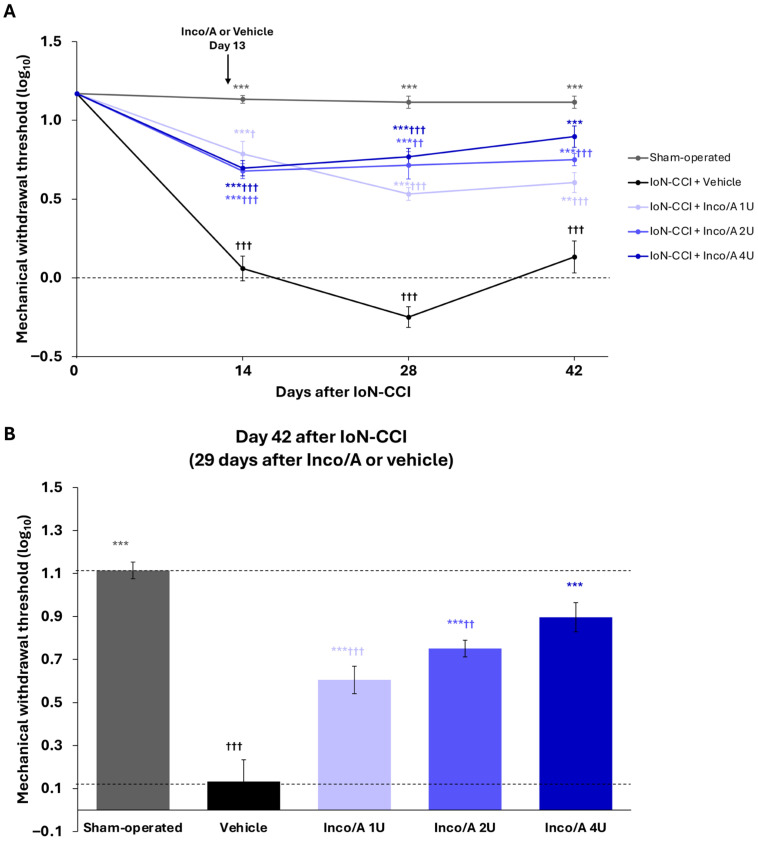
Effect of different doses of incobotulinumtoxinA (Inco/A) in the infraorbital nerve chronic constriction injury (IoN-CCI) model of trigeminal pain in rats (study 1). (**A**) Time course of mechanical withdrawal thresholds for each treatment group. Statistical analysis: repeated measures two-way analysis of variance (ANOVA) with Geisser–Greenhouse correction, followed by Tukey’s multiple comparisons test. (**B**) Dose–response effect on day 42 after surgery (29 days post-treatment). Values shown are mean (SEM). The upper dashed line indicates the mean mechanical withdrawal threshold (MWT) value for sham-operated animals (reflecting absence of pain-related behavior), and the lower dotted line indicates the mean MWT value for IoN-CCI vehicle-treated animals (reflecting mechanical allodynia). Statistical analysis: one-way ANOVA test followed by Tukey’s multiple comparisons test. Values shown are mean (SEM). *n* = 10 per group. ** *p* < 0.01, *** *p* < 0.001 for comparison with vehicle-treated; ^†^ *p* < 0.05; ^††^ *p* < 0.01; ^†††^ *p* < 0.001 for comparison with sham-operated. SEM, standard error of the mean.

IoN-CCI induced robust allodynia-like behavior, as evidenced by markedly lower MWTs in all animals compared with those in the sham-operated group at all post-surgical time points (Figure 2A). On day 42, vehicle-treated animals that had undergone IoN-CCI had the lowest MWT of all the groups, with a log_10_ mean ± SEM MWT of 0.13 ± 0.10. At the same time point, the sham-operated group’s MWT was 1.11 ± 0.04 (*p* < 0.001 vs. vehicle-treated group; Figure 2B).

Treatment with all doses of Inco/A significantly increased the pain threshold of the IoN-CCI model as early as 1 day post-administration, with the effect remaining significant until study end (day 42 = 29 days post-treatment) (two-way ANOVA: F treatment (4, 45) = 111.2, *p* < 0.001; F time (2420, 108.9) = 132.9, *p* < 0.001; F time × treatment (9681, 108.9) = 19.4, *p* < 0.001) (Figure 2A). On day 42, an increasing anti-allodynic effect was observed with increasing doses of Inco/A, with all Inco/A-treated groups demonstrating significantly higher pain thresholds than the vehicle-treated group (*p* < 0.001 for all; one-way ANOVA with Tukey’s multiple comparisons test, F (4, 45) = 31.03, *p* < 0.001).

Animals given 4 U Inco/A demonstrated a pain threshold statistically similar to those in the sham-operated group on day 42, with log_10_ mean ± SEM MWTs of 0.90 ± 0.07 and 1.11 ± 0.04, respectively (*p* = 0.09) (Figure 2B). However, the anti-allodynic effect of 4 U on day 42 did not significantly differ from that of 2 U (*p* = 0.38, two-way ANOVA with Tukey’s multiple comparisons test). Therefore, for subsequent studies, the dose of 2 U Inco/A was selected as the study dose (minimal dose that elicited the greatest anti-allodynic efficacy).

### 3.2. Study 2: Local, Regional, and Systemic Anti-Allodynic Effects of Inco/A

**Figure 3:**
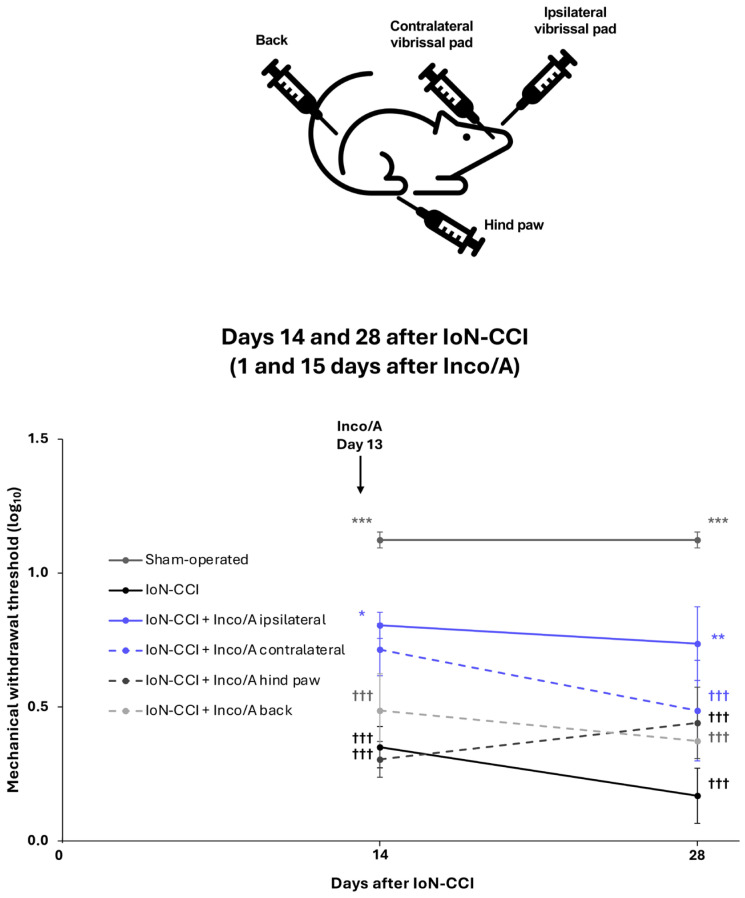
Effect of incobotulinumtoxinA (Inco/A) administration into different body sites in the infraorbital nerve chronic constriction injury (IoN-CCI) model of trigeminal pain in rats (study 2). On day 13 after IoN-CCI induction, 2 U Inco/A was administered subcutaneously to rats in one of four locations: ipsilateral vibrissal pad; contralateral vibrissal pad; hind paw; back. Mechanical withdrawal thresholds (MWTs) were assessed on days 14 and 28 (1 and 15 days post-treatment). Statistical analysis: repeated measures two-way analysis of variance (ANOVA) with Tukey’s test for multiple comparisons. Values shown are mean (SEM). *n* = 8 per group. * *p* < 0.05, ** *p* < 0.01, *** *p* < 0.001 for comparison with IoN-CCI without treatment; ^†††^ *p* < 0.001 for comparison with sham-operated. contra, contralateral; ipsi, ipsilateral; SEM, standard error of the mean.

A significantly higher MWT was observed on day 14 (1 day after treatment) in the animals that had received 2 U Inco/A into the ipsilateral vibrissal pad compared with the untreated animals that had undergone IoN-CCI (log_10_ mean ± SEM MWT 0.81 ± 0.05 and 0.35 ± 0.08, respectively; *p* < 0.05) (Figure 3). Although a trend towards a higher pain threshold was noted in the rats that had received contralateral vibrissal pad injection (log_10_ mean ± SEM MWT 0.71 ± 0.10 on day 14), this effect did not reach statistical significance versus the untreated animals that had undergone IoN-CCI (*p* = 0.15). These findings persisted to day 28 (15 days after treatment), with the ipsilateral effect remaining robust (Inco/A: log_10_ mean ± SEM MWT 0.74 ± 0.14; untreated animals that had undergone IoN-CCI: 0.17 ± 0.10; *p* < 0.01 [two-way ANOVA, F treatment (5, 42) = 14.39, *p* < 0.001]).

The pain thresholds demonstrated by animals given Inco/A in the ipsilateral vibrissal pad were not significantly different from those observed in the sham-operated group on day 14 (log_10_ mean ± SEM MWT 0.81 ± 0.05 and 1.12 ± 0.03, respectively; *p* = 0.27) or day 28 (log_10_ mean ± SEM MWT 0.74 ± 0.14 and 1.12 ± 0.03, respectively; *p* = 0.11). The pain thresholds of animals given Inco/A in the contralateral vibrissal pad did not differ from those of the sham-operated group on day 14 (*p* = 0.07) but were significantly lower on day 28 (*p* < 0.001).

No analgesic effect was detected in the groups injected with Inco/A in the hind paw or back at any time point.

### 3.3. Study 3: Preventive and Therapeutic Anti-Allodynic Effects of Inco/A

**Figure 4:**
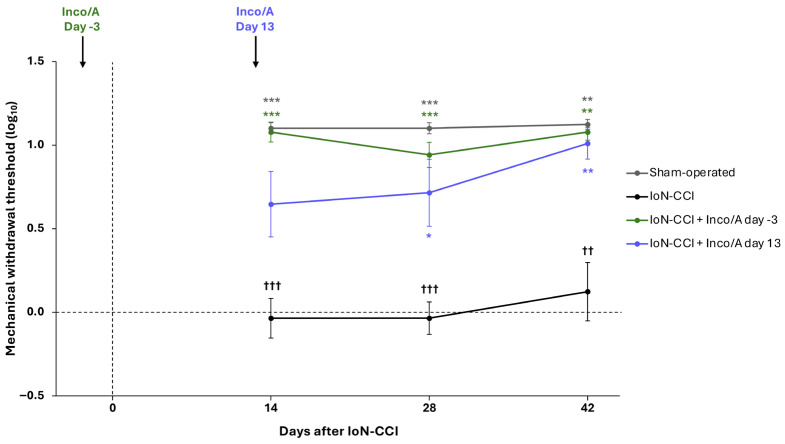
Preventive and therapeutic treatment effects of incobotulinumtoxinA (Inco/A) in the infraorbital nerve chronic constriction injury (IoN-CCI) model of trigeminal pain in rats (study 3). Inco/A (2 U) was administered subcutaneously into the ipsilateral vibrissal pad either 3 days before (day −3; preventive group, green line) or 13 days after (day 13; therapeutic group, blue line) IoN-CCI surgery. Mechanical withdrawal thresholds (MWTs) were assessed on study days 14, 28, and 42 (1, 15, and 29 days post-treatment). Statistical analysis: repeated measures two-way analysis of variance (ANOVA) with Geisser–Greenhouse correction and Tukey’s post hoc test. Values shown are mean (SEM). *n* = 8 per group. * *p* < 0.05, ** *p* < 0.01, *** *p* < 0.001 for comparison with IoN-CCI without treatment; ^††^ *p* < 0.01, ^†††^ *p* < 0.001 for comparison with sham-operated. SEM, standard error of the mean.

A significantly higher sensory threshold was observed on day 14 (1 day after treatment) in the groups treated with 2 U Inco/A on day −3 (i.e., preventive treatment) compared with the untreated animals that had undergone IoN-CCI (Inco/A: log_10_ mean ± SEM MWT 1.08 ± 0.06; no treatment: −0.04 ± 0.12; *p* < 0.001) (Figure 4). In contrast, the group that received 2 U Inco/A on day 13 (therapeutic treatment) showed only a non-significant trend towards improvement versus the untreated animals that had undergone IoN-CCI on day 14 (log_10_ mean ± SEM MWT 0.65 ± 0.20; *p* = 0.05).

There was a significant effect of Inco/A treatment and of time and subject (treatment, *p* < 0.001; time, *p* < 0.05; subject, *p* < 0.001); however, the interaction time x treatment was not significant.

The sensory thresholds achieved by the animals treated with Inco/A were not significantly different from those in the sham-operated group at any time point (days 14, 28, and 42), regardless of the timing of Inco/A administration (day −3 or 13). For the sham-operated group, the log_10_ mean ± SEM MWT was 1.10 ± 0.03 on day 14, 1.10 ± 0.03 on day 28, and 1.12 ± 0.03 on day 42. For the group that received preventive Inco/A (treatment on day −3), log_10_ mean ± SEM MWTs on days 14, 28, and 42 were, respectively, 1.08 ± 0.06, *p* = 0.98 versus the sham-operated group; 0.94 ± 0.07, *p* = 0.27; and 1.08 ± 0.05, *p* = 0.85. For the group that received therapeutic Inco/A (treatment on day 13), log_10_ mean ± SEM MWTs on days 14, 28, and 42 were, respectively, 0.65 ± 0.20, *p* = 0.19 versus the sham-operated group; 0.71 ± 0.20, *p* = 0.30; and 1.01 ± 0.10, *p* = 0.67.

### 3.4. Study 4: Duration of Anti-Allodynic Effects of Inco/A

**Figure 5:**
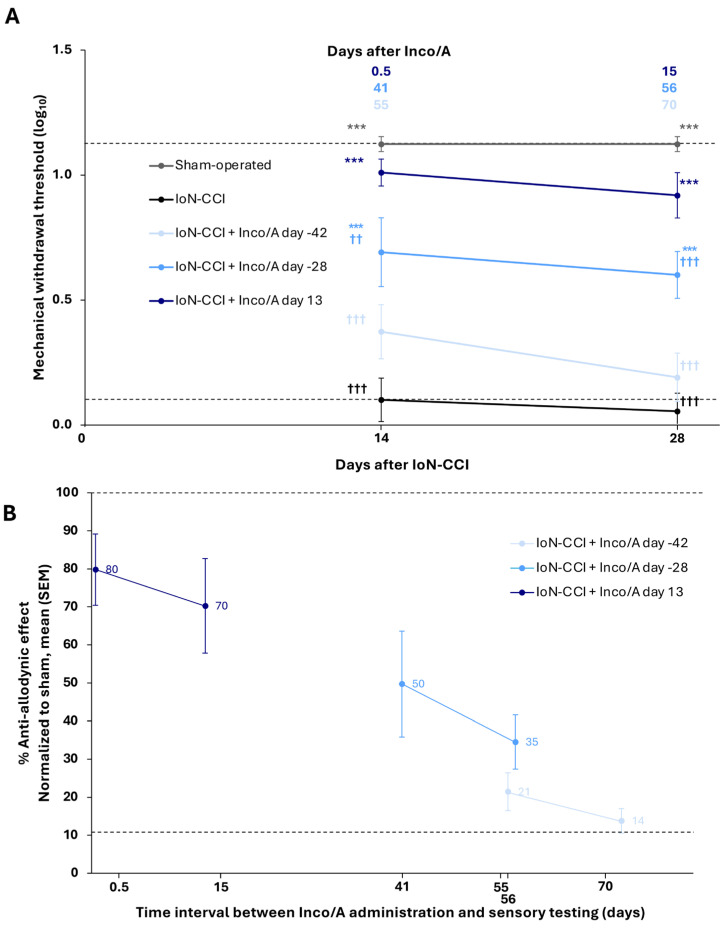
Duration of anti-allodynic effects of incobotulinumtoxinA (Inco/A) in the infraorbital nerve chronic constriction injury (IoN-CCI) model of trigeminal pain in rats (study 4). (**A**) Inco/A (2 U) was administered subcutaneously at three time points (42 or 28 days before, or 13 days after IoN-CCI surgery). Mechanical withdrawal thresholds (MWTs) were measured on post-surgical days 13 and 28, corresponding to up to 70 days post-treatment. (**B**) Anti-allodynic effect of Inco/A calculated using MWTs normalized to those of the sham-operated group (sham-operated group values designated as 100%). The upper dotted line indicates the mean MWT value for sham-operated animals, and the lower dotted line indicates the mean MWT value for IoN-CCI vehicle-treated animals. Statistical analysis: repeated measures two-way analysis of variance (ANOVA) with Tukey’s post hoc test. Values shown are mean (SEM). *n* = 8 per group. *** *p* < 0.001 for comparison with IoN-CCI without treatment; ^††^ *p* < 0.01, ^†††^ *p* < 0.001 for comparison with sham-operated. SEM, standard error of the mean.

Administration of Inco/A 42 days before surgery was not associated with a significant anti-allodynic effect at either of the two post-surgical time points tested (day 13: Inco/A: log_10_ mean ± SEM MWT 0.37 ± 0.11; IoN-CCI and no treatment: 0.10 ± 0.09, *p* = 0.18; day 28: Inco/A: 0.19 ± 0.10; IoN-CCI and no treatment: 0.06 ± 0.07, *p* = 0.80) (Figure 5A). However, administration of Inco/A 28 days before surgery resulted in a significant improvement in MWT at both time points (day 13: Inco/A: log_10_ mean ± SEM, 0.69 ± 0.14; IoN-CCI and no treatment: 0.10 ± 0.09, *p* < 0.001; day 28: Inco/A: 0.60 ± 0.09; IoN-CCI and no treatment: 0.06 ± 0.07, *p* < 0.001). Interestingly, administration of Inco/A on post-surgical day 13 resulted in a strong improvement in mechanical allodynia as early as 4 h post-treatment (day 13: Inco/A: log_10_ mean ± SEM MWT 1.01 ± 0.05; IoN-CCI and no treatment: 0.10 ± 0.09, *p* < 0.001), as well as on day 28 (Inco/A: 0.92 ± 0.09; IoN-CCI and no treatment: 0.06 ± 0.07, *p* < 0.001).

In contrast to administration at both preventive treatment times, only day 13 Inco/A administration (i.e., therapeutic treatment) resulted in sensory threshold levels similar to those seen in the sham-operated group (day 13: Inco/A: log_10_ mean ± SEM MWT 1.01 ± 0.05; sham-operated: 1.12 ± 0.03, *p* = 0.88; day 28: Inco/A: 0.92 ± 0.09; sham-operated: 1.12 ± 0.03, *p* = 0.45). The effect of treatment across all time points was significant (two-way ANOVA, F treatment (4, 35) = 47.84, *p* < 0.001).

The use of normalized MWTs (sham-operated group values designated as 100%) to determine the relationship between timing of Inco/A administration and analgesic effect revealed that maximum efficacy was reached when Inco/A was administered therapeutically (i.e., post-injury). An 80% reduction in pain was achieved on the day of injection and a 70% reduction 15 days later (Figure 5B). However, −28 day preventive regimen demonstrated persistent efficacy, with observable benefits for up to 56 days after treatment (treatment on day −28, testing on day 28).

## 4. Discussion

In this study, the IoN-CCI model of trigeminal pain in rats was employed to evaluate the anti-allodynic efficacy of Inco/A as a potential treatment for TN. We have shown that a 2 U dose of Inco/A elicited a robust anti-allodynic effect in the IoN-CCI rat model and that these effects resulted predominantly from local administration. Both preventive and therapeutic regimens produced sustained anti-allodynic benefits in our assessments, with a significant anti-allodynic efficacy observed even when the administration occurred up to 28 days before surgery.

Surgical induction of IoN-CCI produced mechanical allodynia in the ipsilateral vibrissal pad of rats. This effect persisted from post-surgical day 13 until the end of each study (day 28 or day 42 post-IoN-CCI). In the dose-ranging study, all Inco/A doses administered (1, 2, and 4 U) elicited clear anti-allodynic responses, significantly reducing mechanical allodynia by 1 day post-injection and with a minimum duration of effect of 29 days. These findings align with previous reports of BoNT/A’s antinociceptive properties in animal models of neuropathic pain [31,32,33,34], including the IoN-CCI model [23,24,25,35,36,37], and establish a reliable foundation for assessing the effects of Inco/A on trigeminal pain. Previous studies using the IoN-CCI model and similar doses of BoNT/A in rats (3–10 U/kg [1–3 U per animal]) reduced facial tactile allodynia and cold-evoked facial grooming within a few days, with the effects lasting for more than 2 weeks [24,25,35,36]. When BoNT/A was given before carrageenan, capsaicin, or formalin, it attenuated mechanical and thermal hypersensitivity and reduced late (plasticity-related) pain phases [35,38,39].

In the present study, injection of Inco/A into the ipsilateral vibrissal pad (the side subjected to IoN-CCI) significantly alleviated tactile allodynia on post-surgical days 14 and 28 (1 and 15 days post-treatment, respectively). There appeared to be some short-term alleviation of allodynia when Inco/A was injected into the contralateral vibrissal pad. Given the anatomical proximity of the ipsilateral and contralateral vibrissal pads, this could be explained by local diffusion of the toxin [40]. In contrast, injections into remote sites such as the hind paw or back had no discernible effect on mechanical allodynia. This indicates that local/regional mechanisms are responsible for efficacy and that systemic exposure—for example via the bloodstream—is unlikely to be responsible.

The issue of systemic exposure has been previously discussed. Using both the IoN-CCI model and a cisplatin-induced chemotherapy model in rats, Waskitho et al. [37] reported a decrease in tactile allodynia after injection of BoNT into the contralateral vibrissal pad. They also performed a mouse bioassay and concluded that approximately 18% of the BoNT injected into the vibrissal pad was found in the circulation. However, it is important to note that Waskitho et al. [37] administered BoNT at substantially higher doses than in our study. In the allodynia study, Waskitho et al. [37] injected 10 times the minimal lethal dose of BoNT/A intradermally into rats’ vibrissal pads. In the mouse bioassay study, they injected 5000 times the minimal lethal dose intradermally into a rat’s vibrissal pad, collected serum from the rat 5 h post-injection, and inoculated serial dilutions of the serum intraperitoneally into mice [37]. In the current study, 2 U of Inco/A was injected in a volume of 5 µL for the majority of analyses. Assuming complete systemic distribution, the theoretical blood concentration would be roughly 0.36 U per 15 mL (the average blood volume of a rat; 18% of Inco/A in the circulation), or 0.00012 U per 5 µL. This represents an approximate 17,000-fold dilution relative to the injected concentration. Naturally, local dilution also occurs at the injection site but to a much lesser extent.

Peripheral BoNT/A injections can lead to limited systemic and trans-synaptic transport in rats (as evidenced by the presence of BoNT/A in blood or labeling in contralateral ganglia or muscles), but exposure is low and not typically associated with muscle weakness, supporting the view that analgesia is largely mediated by local neuronal uptake and transport within the trigeminal system [41].

Taken together, these findings suggest that systemic distribution of Inco/A, if present, plays only a minor role in its analgesic action, whereas local and possibly intra-neuronal transport are the dominant mechanisms. This is supported by the lack of motor deficits after subcutaneous injection.

Inco/A also conferred benefits when administered both preventively and therapeutically, with effects that were not only robust but also long-lasting. Comparable anti-allodynic efficacy was observed whether Inco/A was administered 3 days before or 13 days after IoN-CCI induction, supporting its utility in both preventive and therapeutic treatment paradigms. Notably, anti-allodynic effects were still observed 56 days after Inco/A administration (when given 28 days before injury and assessed 28 days after), indicating a substantial duration of action. To our knowledge, this is the first study evaluating the effect of BoNT/A treatment in an IoN-CCI model for an extended period. Our observations are in line with the findings of Li et al. [42] regarding a post-operative pain model in which BoNT/A was administered 1 day or 2 days before pain induction in rats. The long-lasting effect observed in our study is translationally relevant; clinical trials of BoNT for TN generally report onset of the analgesic action within 1–2 weeks and duration of effects of approximately 3–6 months, with considerable improvements in QoL [43,44,45,46,47,48,49,50].

Most clinical studies to date have investigated onabotulinumtoxinA. However, available clinical data suggest that Inco/A may offer comparable efficacy and distinct advantages [51]. The two products contain the same active 150 kDa neurotoxin molecule, with onabotulinumtoxinA containing additional neurotoxin-complexing proteins that have no effect on the neurotoxin’s mode of action [52,53]. The absence of these proteins in Inco/A reduces the overall foreign protein load, potentially lowering immunogenicity and the likelihood of neutralizing antibody development. Considering the recurrent and severe nature of pain attacks in TN, the findings presented here support the potential of Inco/A as both a preventive and a therapeutic treatment option. However, it is important to note that the duration of BoNT/A’s effects in rodents differs from that in humans. This likely reflects species-specific neuronal biology and toxin handling, with rodents exhibiting faster and more robust peripheral nerve recovery than humans and shorter persistence of BoNT/A activity [54,55,56,57].

The mechanism of action of BoNT/A in pain modulation, though incompletely elucidated, has been documented in several comprehensive reviews [43,44,58]. BoNT/A modulates pain by primarily binding to synaptic vesicle glycoprotein 2 receptors at peripheral nerve terminals, where it is internalized and blocks the release of key neurotransmitters involved in nociceptive transmission, such as substance P, calcitonin gene-related peptide, and glutamate. BoNT/A also decreases the expression of pronociceptive genes, including those encoding ion channels and cytokines in damaged neurons, thereby limiting neurogenic inflammation [44]. Importantly, the effects of BoNT/A are not restricted to peripheral nerve endings but extend along the entire primary sensory neuron. There is accumulating evidence for retrograde axonal transport of BoNT/A within sensory neurons, enabling the toxin to reach the presynaptic terminals in the dorsal horn of the spinal cord [43,58]. Additionally, BoNT/A disrupts synaptic vesicle transport, which impairs trafficking and surface expression of pain receptors and numerous ion channels. This reduces the overall excitability of sensory neurons and leads to attenuation of pain transmission. As a result, both peripheral and, indirectly, central sensitization are effectively attenuated or prevented [43,59].

In the context of TN, pain generation in both classical and secondary TN is fundamentally driven by demyelination and subsequent axonal changes, which lower neuronal excitability thresholds and promote ectopic firing and ephaptic crosstalk between demyelinated fibers. These processes are further characterized by abnormal expression and function of sodium channels (NaV1.7, NaV1.3, NaV1.8) and voltage-gated potassium channels, disrupting the stability of resting membrane potentials and predisposing affected neurons to spontaneous and evoked hyperexcitability [43]. BoNT/A exerts therapeutic effects in this setting by inhibiting soluble N-ethylmaleimide-sensitive factor attachment protein receptor-dependent neurotransmitter release at presynaptic terminals and may counteract the abnormal sodium and potassium channel-driven excitability, directly targeting the heightened synaptic activity and neurogenic inflammation that maintain neuropathic pain in demyelinated trigeminal pathways [43].

This study has several limitations. Although the IoN-CCI animal model is validated for the neuropathic pain associated with trigeminal nerve damage, it is not an optimal model for secondary or idiopathic TN. Post-surgical pre-treatment testing was not performed, although baseline mechanical thresholds were assessed prior to surgery, and vehicle-treated animals that had undergone IoN-CCI served as the surgical control group and demonstrated robust and persistent mechanical allodynia consistent with the established IoN-CCI model. In addition, the study assessed mechanical pain thresholds evoked using von Frey hairs, which do not capture the full impact of pain on the QoL of human patients. Mechanical allodynia assessed by von Frey testing was selected as the primary outcome measure of the study to reflect the pain attacks triggered by usually non-painful events (e.g., chewing, brushing teeth or facial touch) but does not consider temperature change. Assessment of thermal sensitivity would have required specialized equipment that was not available at the study site. Similarly, alternative approaches, including longitudinal and non-evoked pain readouts, may provide more comprehensive insights into the therapeutic impact of BoNT/A on patient well-being. Lastly, only male rats were studied. Given that TN affects more women than men, there is an unmet need for preclinical studies using female animal models.

## 5. Conclusions

Inco/A (Xeomin^®^) is highly effective in reducing mechanical allodynia in the IoN-CCI model of trigeminal pain in rats. The analgesic effects are predominantly local, with a negligible role for systemic contribution. This suggests that the analgesia is effected via local neuronal uptake and intra-neuronal distribution, rather than systemic or remote effects, and is therefore consistent with a favorable safety profile. Both preventive and therapeutic regimens are beneficial, with marked long-lasting efficacy. Remarkably, benefits were observed even when Inco/A was administered up to 28 days before injury, indicating a substantial preventive window. Given the reliability, magnitude, and duration of the anti-allodynic effects of Inco/A in this validated preclinical model, these findings strongly support further investigation and clinical translation for both prevention and management of TN and potentially other related neuropathic pain syndromes.

## Figures and Tables

**Table 1:** Filament number and bending forces of the monofilaments from the sensory testing kit used.

Filament Number	Bending Force (Grams)
1.65	0.008
2.36	0.02
2.44	0.04
2.83	0.07
3.22	0.16
3.61	0.4
3.84	0.6
4.08	1.0
4.17	1.4
4.31	2.0
4.56	4.0
4.74	6.0
4.93	8.0
5.07	10.0
5.18	15.0
5.46	26.0
5.88	60.0
6.10	100
6.45	200
6.65	300

## Data Availability

The research questions, study designs and analysis plans were prepared before study execution. No registrations in public repositories were made. All raw data are available in the results database Quattro at Merz Therapeutics.

## Supplementary Materials

The following supporting information can be downloaded at: https://www.mdpi.com/article/10.3390/biomedicines14051175/s1, Table S1: Change in body weight of animals throughout Study 1; Table S2: Change in body weight of animals throughout Study 2; Table S3: Change in body weight of animals throughout Study 3; Table S4: Change in body weight of animals throughout Study 4.

## Abbreviations

The following abbreviations are used in this manuscript:

TN	trigeminal neuralgia
BoNT/A	botulinum neurotoxin type A
Inco/A	incobotulinumtoxinA
MS	multiple sclerosis
QoL	Quality of life
IoN-CCI	infraorbital nerve chronic constriction injury
HDB	HD Bioscience
MWT	mechanical withdrawal threshold
ANOVA	analysis of variance
SEM	standard error of the mean
