# Supplementary material for: Effects of IncobotulinumtoxinA in the Infraorbital Nerve Chronic Constriction Injury Model of Trigeminal Pain in Rats
Source: Biomedicines. 2026 May 21;14(5):1175. doi: 10.3390/biomedicines14051175 (PMC13204939; doi:10.3390/biomedicines14051175)
Supplement: Supplementary file 1 [file biomedicines-14-01175-s001.zip › biomedicines-4214685-supplementary.pdf]

biomedicines

MDPI

Supplementary Tables

Table S1. Change in body weight of animals throughout Study 1.

Group	Day 1	Day 14	Day 28	Day 42
Sham operated animals	268.1 (3.1)	377.3 (6.6)	446.2 (10.7)	490.1 (10.7)
IoN-CCI + Vehicle	268.2 (3.2)	361.2 (4.5)	429.2 (6.3)	477.8 (7.9)
IoN-CCI + 1 U Inco/A	268.3 (3.2)	362.2 (5.8)	428.3 (8.3)	473.9 (9.0)
IoN-CCI + 2 U Inco/A	269.0 (3.3)	362.0 (4.7)	418.8 (6.0)	467.0 (7.7)
IoN-CCI + 4 U Inco/A	269.2 (3.4)	358.2 (6.8)	406.5 (8.0)	453.2 (8.9)

Data are reported as mean (standard error of the mean) in grams.
Inco/A, incobotulinumtoxinA; IoN-CCI, infraorbital nerve chronic constriction injury

Table S2. Change in body weight of animals throughout Study 2.

Group	Day 0	Day 14	Day 28
Sham operated animals	249.4 (4.7)	358.4 (14.8)	435.3 (22.2)
IoN-CCI	249.0 (3.8)	356.8 (7.5)	430.0 (10.7)
IoN-CCI + 2 U Inco/A ipsilateral	248.6 (3.4)	348.4 (7.2)	417.0 (13.0)
IoN-CCI + 2 U Inco/A contralateral	248.7 (3.4)	358.2 (6.8)	430.4 (10.9)
IoN-CCI + 2 U Inco/A hind paw	249.7 (3.7)	357.4 (7.7)	428.7 (10.9)
IoN-CCI + 2 U Inco/A back	249.3 (3.3)	358.6 (9.0)	421.2 (13.0)

Data are reported as mean (standard error of the mean) in grams.
Inco/A, incobotulinumtoxinA; IoN-CCI, infraorbital nerve chronic constriction injury

Table S3. Change in body weight of animals throughout Study 3.

Group	Day -3	Day 0	Day 14	Day 28	Day 42
Sham operated animals	262.7 (2.6)	289.9 (1.3)	388.9 (4.3)	457.9 (4.9)	507.0 (6.2)
IoN-CCI	263.4 (2.0)	288.4 (2.5)	371.3 (6.0)	438.9 (9.0)	482.8 (12.8)
IoN-CCI + 2 U Inco/A on day -3	264.0 (1.7)	284.6 (2.1)	365.6 (5.6)	435.9 (8.0)	484.9 (11.8)
IoN-CCI + 2 U Inco/A on day 13	264.0 (1.5)	285.0 (2.6)	367.8 (5.0)	432.7 (7.7)	479.1 (9.4)

Data are reported as mean (standard error of the mean) in grams.
Inco/A, incobotulinumtoxinA; IoN-CCI, infraorbital nerve chronic constriction injury

 https://doi.org/10.3390/biomedicines14051175

Table S4. Change in body weight of animals throughout Study 4.

Group	Day -42	Day -35	Day -28	Day -21	Day -14	Day -7	Day 0	Day 7	Day 13	Day 21	Day 28
Sham operated animals	311.9 (5.4)	360.5 (7.7)	402.8 (9.5)	436.2 (11.0)	466.8 (11.8)	494.4 (12.6)	517.2 (14.0)	526.4 (14.8)	541.6 (15.2)	561.1 (14.8)	581.1 (16.7)
IoN-CCI	311.9 (6.2)	356.6 (7.4)	402.1 (9.3)	434.3 (10.3)	464.3 (11.8)	487.1 (13.7)	511.5 (13.7)	514.9 (14.7)	531.1 (15.2)	544.9 (16.9)	580.7 (18.9)
IoN-CCI + 2 U Inco/A on day -42	310.7 (4.6)	352.5 (7.7)	386.5 (9.2)	418.8 (10.6)	448.2 (12.0)	470.4 (13.6)	495.3 (15.5)	504.7 (16.0)	522.6 (16.3)	543.6 (15.6)	567.2 (17.1)
IoN-CCI + 2 U Inco/A on day -28	311.8 (4.5)	357.1 (6.2)	399.2 (8.6)	420.1 (9.6)	446.4 (12.4)	468.0 (16.0)	490.7 (16.6)	494.1 (16.6)	514.2 (15.6)	543.8 (17.2)	565.3 (17.0)
IoN-CCI + 2 U Inco/A on day 13	312.9 (4.6)	363.7 (6.5)	402.6 (8.0)	422.7 (10.9)	453.8 (11.9)	474.4 (16.0)	495.5 (16.5)	505.4 (14.5)	526.5 (15.6)	540.6 (16.2)	557.5 (16.0)

Data are reported as mean (standard error of the mean) in grams.
Inco/A, incobotulinumtoxinA; IoN-CCI, infraorbital nerve chronic constriction injury

https://doi.org/10.3390/biomedicines14051175
